# Validating a dimension of doubt in decision-making: A proposed endophenotype for obsessive-compulsive disorder

**DOI:** 10.1371/journal.pone.0218182

**Published:** 2019-06-13

**Authors:** Tanya Marton, Jack Samuels, Paul Nestadt, Janice Krasnow, Ying Wang, Marshall Shuler, Vidyulata Kamath, Vikram S. Chib, Arnold Bakker, Gerald Nestadt

**Affiliations:** 1 Department of Neuroscience, The Johns Hopkins University School of Medicine, Baltimore, Maryland, United States of America; 2 Department of Psychiatry and Behavioral Sciences, The Johns Hopkins University School of Medicine, Baltimore, Maryland, United States of America; 3 Department of Biomedical Engineering, The Johns Hopkins University School of Medicine, Baltimore, Maryland, United States of America; 4 Kennedy Krieger Institute, Baltimore, Maryland, United States of America; Academic Medical Center, NETHERLANDS

## Abstract

Doubt is subjective uncertainty about one’s perceptions and recall. It can impair decision-making and is a prominent feature of obsessive-compulsive disorder (OCD). We propose that evaluation of doubt during decision-making provides a useful endophenotype with which to study the underlying pathophysiology of OCD and potentially other psychopathologies. For the current study, we developed a new instrument, the Doubt Questionnaire, to clinically assess doubt. The random dot motion task was used to measure reaction time and subjective certainty, at varying levels of perceptual difficulty, in individuals who scored high and low on doubt, and in individuals with and without OCD. We found that doubt scores were significantly higher in OCD cases than controls. Drift diffusion modeling revealed that high doubt scores predicted slower evidence accumulation than did low doubt scores; and OCD diagnosis lower than controls. At higher levels of dot coherence, OCD participants exhibited significantly slower drift rates than did controls (q<0.05 for 30%, and 45% coherence; q<0.01 for 70% coherence). In addition, at higher levels of coherence, high doubt subjects exhibited even slower drift rates and reaction times than low doubt subjects (q<0.01 for 70% coherence). Moreover, under high coherence conditions, individuals with high doubt scores reported lower certainty in their decisions than did those with low doubt scores. We conclude that the Doubt Questionnaire is a useful instrument for measuring doubt. Compared to those with low doubt, those with high doubt accumulate evidence more slowly and report lower certainty when making decisions under conditions of low uncertainty. High doubt may affect the decision-making process in individuals with OCD. The dimensional doubt measure is a useful endophenotype for OCD research and could enable computationally rigorous and neurally valid understanding of decision-making and its pathological expression in OCD and other disorders.

## Introduction

Making decisions promptly and accurately is fundamental to adaptive functioning. Individual differences in this ability may reflect differences in underlying physiological processes and related cognitive traits, and in extreme cases may give rise to specific psychopathology. A rigorous scientific understanding of this dimension and its pathological expression requires the characterization of the mechanisms of decision-making, their sources of variability, and their points of vulnerability.

An important component of the decision-making process is having confidence in the information necessary to make a decision. Doubt has been described as a lack of subjective certainty about, and confidence in, one’s perceptions and internal states [1]. Doubt is a prominent feature in many patients with obsessive-compulsive disorder (OCD), in whom doubt has been described as an inability to “experience a sense of conviction”, to put closure on experience, or to generate the normal “feeling of knowing” [2,3]. Several studies have found that OCD is associated with lack of confidence in one’s own memory, attention, and perception [4–6], and studies using cognitive assessment have found that individuals with OCD or high compulsivity require more time and experience more uncertainty in decision-making tasks [7, 8]. Recently, Banca et al. asked subjects to determine the direction of a collection of pseudo-randomly moving dots and found that, when in conditions of low-uncertainty, individuals with OCD were slower in making their decisions [9]. Hauser et al., employing a similar task design, replicated several of these findings in perceptual decision-making, and identified an additional metacognitive uncertainty in ‘compulsive’ subjects when compared to controls [10, 11]. It has been proposed that the lack of certainty when assimilating information contributes to decision-making difficulties experienced by many individuals with OCD [12].

However, we know of no evidence that a dimensional cognitive trait underlies the decision-making process in unaffected individuals, or that a disrupted decision-making process in OCD is related to a dimensional trait of doubt. Furthermore, we know of no evidence linking variation in this dimensional trait, or evidence linking it or OCD to a dimension of neurophysiological variability. A reliable and valid dimensional measure of doubt would provide a means of investigating these hypotheses.

A plausible model of doubt is that it represents the rate of internal computations that determine decisions and the neurophysiological basis for these computations. A valid neurophysiological and computational model of decision-making would therefore define cognitive traits as the consequence of variability in the processes by which individuals integrate information and direct their behavior. More specifically, individual variability can be explained by neurophysiological parameter differences in the computations underlying decision-making. Here, we aim to measure doubt and determine whether variation in this trait reflects variability in underlying neurophysiological processes that reflect computational parameters of decision-making.

The studies presented in this paper describe the development of a measure of doubt and address the hypotheses that a) individuals vary along a cognitive dimension, doubt; b) this dimension is relatable to the computational parameters that describe how external evidence is accumulated for decision-making under uncertainty; and c) extremes on this dimension are observed in patients with OCD. To test these hypotheses, we developed a multi-item questionnaire that measures doubt, examined its dimensionality, and evaluated its psychometric properties in individuals who completed an internet survey. We also compared doubt scores in individuals with OCD and in community volunteers. We then evaluated performance on a decision-making task in individuals with and without OCD, as well as those scoring high or low on doubt, using a random dot motion paradigm to assess computational parameters based on the “drift diffusion” model of decision-making [13].

## Materials and methods

### Development of the Doubt Questionnaire (DQ)

This study was approved by the Johns Hopkins Medical Institutions Institutional Review Board IRB2 (Approval number NA_00040491). Written consent was obtained.

The initial item pool for the DQ was generated by clinicians (Drs. Nestadt and Krasnow) expert in the evaluation and diagnosis of OCD and anxiety disorders in patients and in participants in family and genetic studies of OCD. The items were devised to capture the experience of doubt in several domains, including memory, decisions, task accuracy and completion, visual perception, and auditory perception. After input from other clinicians and researchers, a preliminary version of the instrument was developed, containing 21 items, including two items from the “Doubts about actions” subscale of the Multidimensional Perfectionism Scale (“Even when I do something very carefully, I often feel that it is not quite done right” and “I usually have doubts about the simple everyday things I do” [14]. After completion by, and feedback from, 10 volunteers, the wording of several items was modified to improve clarity, and three items relating to auditory perceptions were deleted, due to potential confusion with the perceived difficulty hearing. The final Doubt Questionnaire (DQ) version included 18 statements, each rated on a five-point Likert scale ranging from “Strongly Disagree” (rating = 1) to “Strongly Agree” (rating = 5). After reverse-coding item 3 (“I trust my own intuition”), the total doubt score was calculated by summing the scores for each item; with scores ranging from 18–90 (Table A in S1 Table). The total doubt score is derived by summing the ratings for each item, with item 3 being reverse-coded.

### Internet surveys

An internet form of the DQ was created on the Qualtrics survey platform (https://www.qualtrics.com). In the first phase, there were two separate postings of the questionnaire. First, a link was made available on a public Facebook page (https://www.facebook.com). Participation was anonymous, and no feedback was provided to the N = 67 participants. Second, the electronic questionnaire was posted online on the OCD Research webpage, within the protected Johns Hopkins University server. Participation was anonymous, but the N = 85 participants were given feedback on their scores, based on the mean and standard deviation of the scores on the first posting. Combining the two samples, there were 152 participants in this first phase.

In addition to completing the Doubt Questionnaire, participants in the internet postings were asked to complete a doubt item investigated in the development of the Yale-Brown Obsessive Compulsive Scale (YBOCS) [15]: “After you complete an activity, do you doubt whether you performed it correctly? Do you doubt whether you did it at all? When carrying out routine activities, do you feel you don’t trust your senses (that is, what you see, hear, or touch)?” The YBOCS doubt item was rated on a 5-point scale: no doubt, mild, moderate, severe, or extreme doubt.

The 152 individuals in this first phase ranged in age from 17–83 years (mean 42.5, SD = 14.6 years); 46 males, 95 were females, and 11 did not provide their gender. The Student t-test was used to compare doubt scores in men and women, and analysis of variance was used to compare total DQ score across YBOCS doubt ratings. Factor analysis on the 18-item DQ was performed using varimax rotation, and the scree plot was used to estimate the number of factors. We also conducted parallel analysis as an alternative, statistical approach for determining the number of components, using an SPSS program developed for this purpose [16].

In the second (test-reliability) phase, students or staff who responded to a request for study participants on the Johns Hopkins University web-based “bulletin board” completed the DQ at two time points, at least one month apart (range, 28–57 days; mean = 34.0 days, SD = 3.5). There were 158 participants in this phase (56 men, 102 women); their ages ranged from 17–59 years (mean 24.7, SD = 9.2 years). The intraclass correlation coefficient was used to evaluate the test-retest reliability for the total doubt score.

### Doubt scores in OCD and comparison groups

A total of 67 OCD patients, diagnosed according to DSM-IV criteria [17], were recruited from the JHU OCD and Anxiety Disorders outpatient clinics, and from individuals participating in an ongoing genetic study of OCD. In addition, 27 community volunteers were recruited as a comparison group. Participants completed the DQ. The mean age of participants was 37.7 years (SD = 15.2; range 12–74 years); 63 were female, 17 were male, and 14 had missing information on gender). The Student t-test was used to compare the mean scores between the OCD cases and controls. In addition, receiver operating characteristic (ROC) analysis was performed to determine the sensitivity and specificity of the DQ doubt score for discriminating between the two groups, and to estimate the area under the sensitivity vs. (1-specificity) curve.

### Behavioral task performance

Seventy individuals (26 OCD participants and 44 controls) were recruited from the JHU OCD and Anxiety Disorders Clinics, from an ongoing genetic study of OCD, and from respondents to postings on JHMI and JHU student and staff web sites, for the behavioral task. Forty-six of these participants also completed the DQ (12 OCD participants and 34 controls).

To evaluate decision-making in these individuals, the random-dot motion task (RDMT) was used [18, 19], as adapted from Banca et al. [9], with the approval and assistance of the study authors. In brief, participants viewed a cloud of dots moving within a borderless circle on the screen. Participants were asked to determine whether the dot cloud appeared to be moving to the right or left, pressing ‘S’ for left and ‘K’ for right on the keyboard using their index fingers. Two sets of 500 dots were presented: the ‘coherent set', in which dots moved coherently, and the ‘random set', in which dots moved randomly. In order to cover a wide range of individual detection thresholds and to represent conditions of varying uncertainty, different motion coherence levels were defined by varying the proportion of dots in the ‘coherent set’. Coherence levels included were 0.025, 0.05, 0.1, 0.15, 0.25, 0.45, and 0.7. Each trial was followed by an inter-trial fixation cross, centered in the middle of the screen, varying between 0.5 and 1 second in duration. The dot cloud was displayed for a maximum duration of 10 seconds, and ceased following a response. Monetary feedback (+$1.00 or −$1.00) indicated whether the response was correct or incorrect.

The task consisted of a practice session and performance under three separate conditions. The first condition included seven coherence levels with monetary feedback. The second condition included six coherence levels and assessed subjective certainty following the decision. However, our results suggest that high were directed to record their level of certainty based on the decision they made on the dot motion. They completed this on a computerized scale ranging from 1 (low certainty) to 7 (high certainty). The third condition, with six coherence levels, introduced a monetary penalty for slow responses, as well as a monetary incentive for fast responses, individualized for reaction time to measure the speed-accuracy trade-off. A response time (RT) greater than 1 standard deviation above the individual’ average RT, calculated from the first condition, was penalized $2.00. Participants received increasing monetary feedback for faster responses and were told that they would receive a proportion of their rewards after completion of the experiment. The primary outcome measures were accuracy, RT, and subjective certainty ratings.

As in Banca et al. [9], hierarchical drift diffusion modelling (HDDM) was used to test the difference of response strategies between groups. In this model, each choice is represented as a diffusion towards an upper and lower decision boundary. When the accumulated noisy evidence reaches one of these two boundaries over time, the decision is made and the respective response initiated [19]. HDDM simultaneously accounts for the proportion of correct and incorrect trials and the respective reaction time (RT) distributions across conditions, considering the latter a result of underlying latent parameters of a decision-making model. It further estimates the posterior probability density of the diffusion model parameters, by using Markov chain Monte Carlo simulation, generating group data, while accounting for individual differences. The estimated model parameters are the drift rate (the speed of the evidence accumulation process towards either boundary, or the quality of the accumulated evidence); and the decision threshold (the distance between the two boundaries, or the amount of evidence accumulated [20].

## Results

### Distribution and reliability of Doubt Questionnaire (DQ) scores in Internet participants

**Fig 1:**
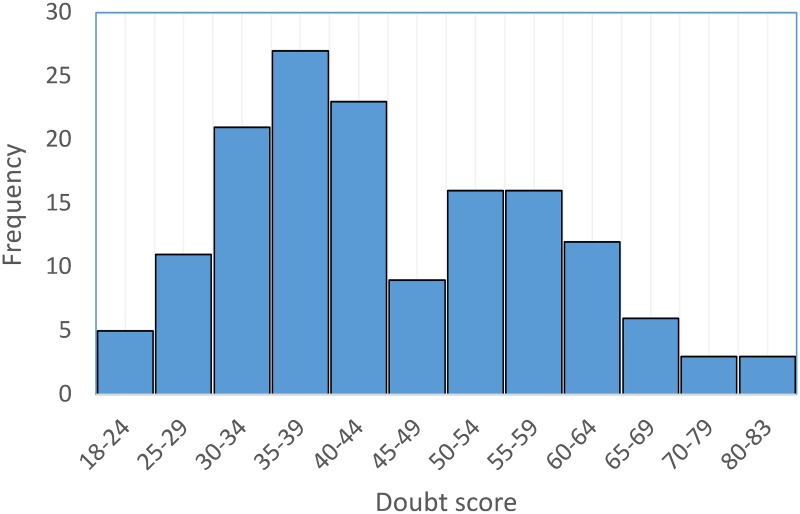
Distribution of doubt scores in internet participants (N = 152).

In the first internet phase, completed by 152 participants, scores on the DQ ranged from 18 to 83 (mean 44.8, SD = 13.6) (Fig 1). The value of the kurtosis statistic (-0.19, SE = 0.39) indicates that the scores cluster less about the center and have thicker tails than a normal distribution, to a slight degree. The value of the skewness statistics (0.52, SE = 0.20) indicates a moderate departure from symmetry in this sample, with a longer right tail compared to a normal distribution (Fig 1). The mean DQ score was similar in men (mean, 45.9; SD = 14.0; range 18–81) and women (mean, 44.9; SD = 13.6; range, 20–83; t_139_ = 0.40, p = 0.69). The DQ score was moderately negatively correlated with age (Pearson r = -0.24, p = 0.003).

The doubt scale had high overall inter-item reliability, with the Spearman-Brown Coefficient = 0.89, and Cronbach’s α = 0.91; this value did not appreciably change, ranging from 0.90–0.91, after individually deleting each item.

Five components, with eigenvalues >1.0, were extracted in the factor analysis of the items. The first component, with eigenvalue = 7.53, explained 41.8% of the variance; the next four components explained 7.9%, 7.2%, 6.0%, and 5.7% of the variance, respectively; the cumulative variance explained was 68.7%. The scree plot suggested a single factor structure for the items in the DQ (Figure A in S1 Fig). Eight items had loadings >0.70; seven items had loadings ranging from 0.50–0.69; and three items had loadings <0.50. Using the RAWPAR algorithm provided by O’Connor [16], we conducted a Monte-Carlo simulation to determine the number of factors to extract, specifying principal components analysis, 1000 parallel data sets, and permutations of the raw data set. Only the first eigenvalue based on the raw data exceeded the 95% percentile of the random-generated eigenvalue distribution, consistent with a single factor underlying the DQ items.

DQ scores were correlated with the YBOCS doubt rating, with mean doubt scores increasing from 37.1 in those rated as having no doubt; 48.5 in those with mild doubt; 56.4 in those with medium doubt; 66.7 in those with severe doubt; and 72.3 in those with extreme doubt (F_4;147_ = 37.6, p<0.001).

In the 158 test-retest participants, the mean doubt scores were 53.7 (SD = 13.4) and 54.0 (SD = 13.2) at first and second completions, respectively. There was an inverse relationship between age and DQ score at the first assessment (Pearson r = -0.23, p<0.01). The mean difference in doubt scores between the two periods was 0.34 (SD = 7.2). The one-month test-retest reliability of the DQ was excellent (intraclass correlation coefficient = 0.86, p<0.001).

### Doubt scores in OCD and comparison participants

**Fig 2:**
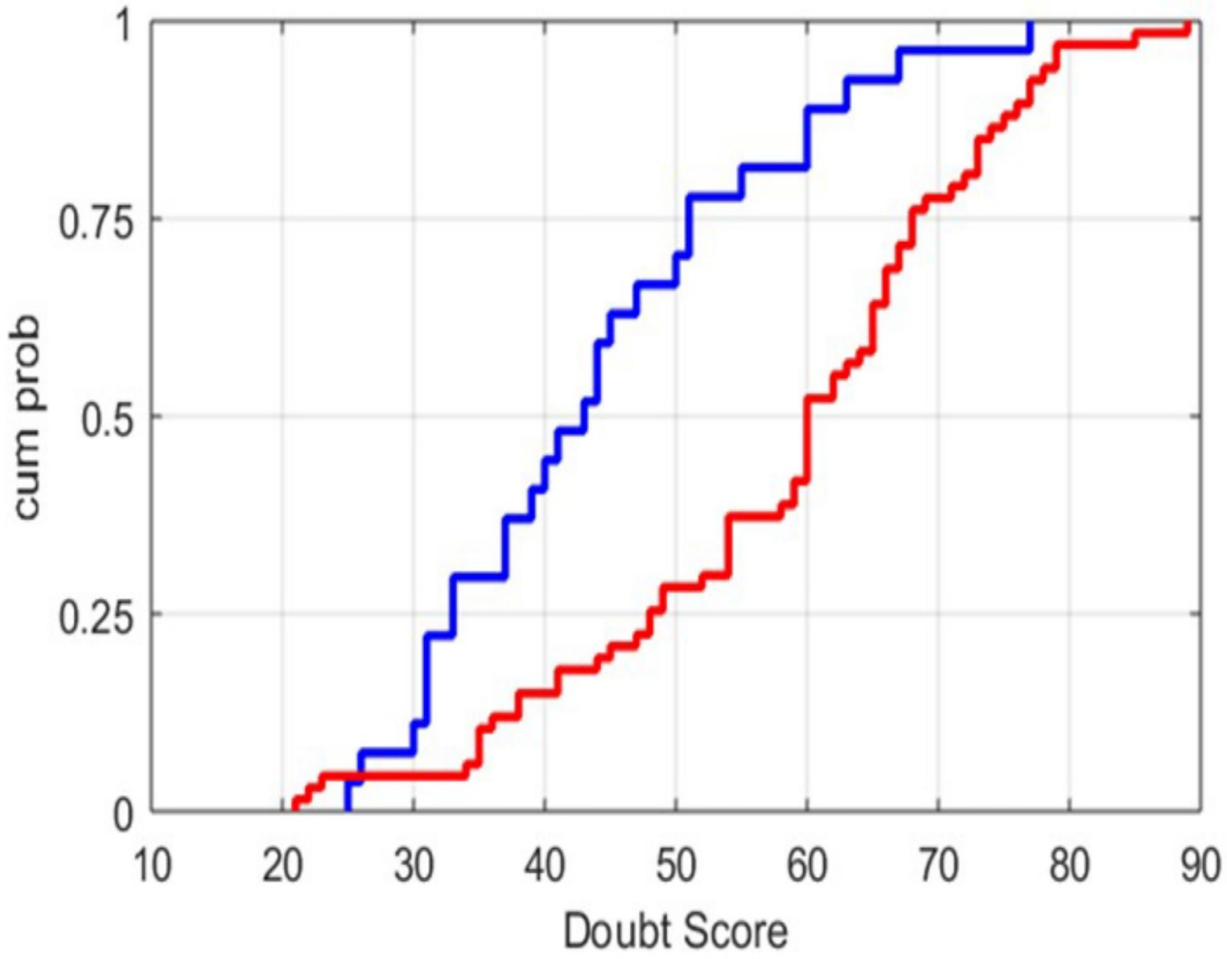
Cumulative probability distributions of doubt scores in OCD and non-OCD participants. OCD (red color; N = 67); non-OCD participants (blue color; N = 27). Participants with OCD exhibited significantly higher doubt scores (rank sum test, p<0.0001).

As described above, 67 OCD patients and 27 non-OCD participants completed the DQ in this sample. In the non-OCD group, the mean doubt score was 44.1 (SD = 13.2; range 25–77), which was not substantially different from the distribution in the internet respondents. In contrast, in OCD cases, the mean doubt score was 58.4 (SD = 15.4; range 21–89), significantly greater than in the internet respondents (t_88_ = 4.09, p<0.001). The distributions of doubt scores between the OCD and comparison groups were significantly different (rank sum test, p<0.0001) (Fig 2). Using a doubt score of 60 and above as the threshold for “high doubt” (i.e., greater than approximately 1 SD above the mean of doubt scores in the internet participants), 39 (58%) of the OCD participants, compared to 5 (19%) of the comparison group, had a high doubt score (χ^2^_1_ = 12.2, p<0.001). From the ROC analysis, the estimated area under the sensitivity vs. (1-sensitivity) curve was 0.76 (95% CI = 0.66–0.87), p<0.001 (Figure B in S2 Fig).

### Decision-making task performance

#### Comparison of OCD cases and controls

**Fig 3:**
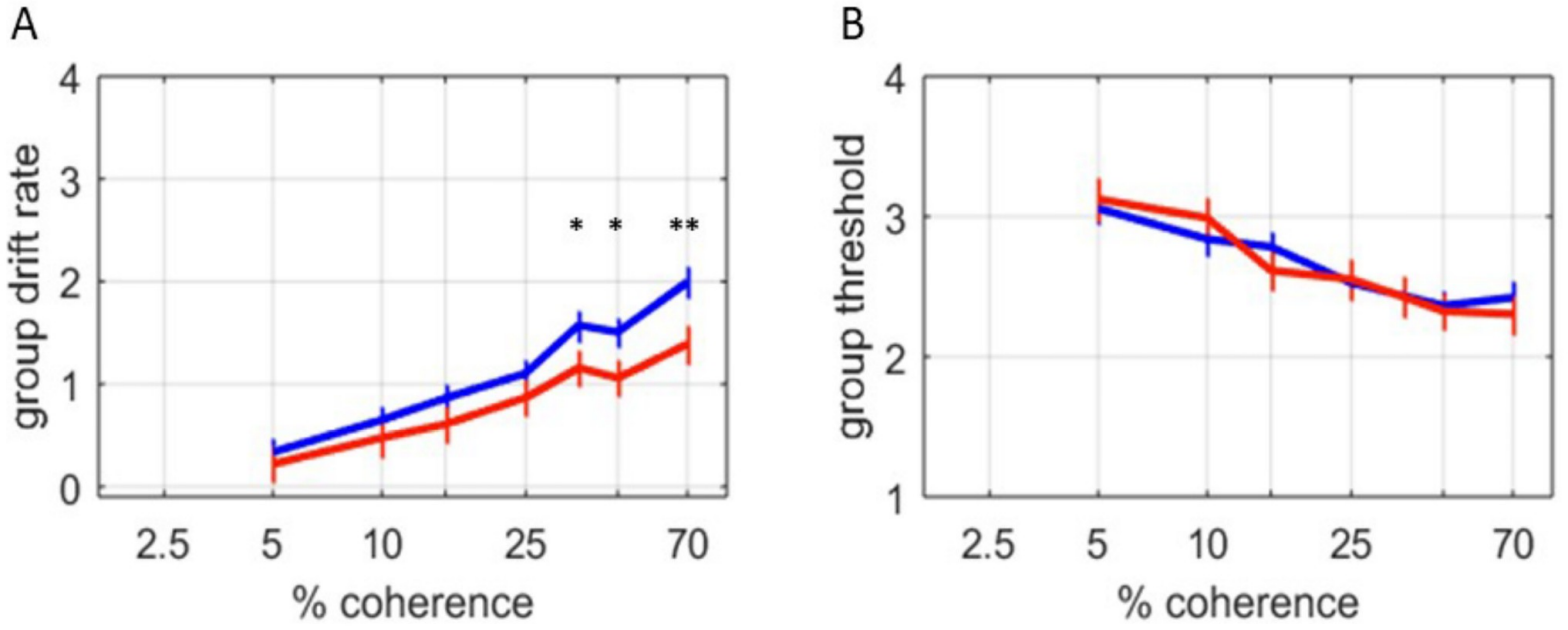
Behavioral task performance in OCD cases and controls. A) A posterior distribution of group mean drift rate was calculated using HDDM, and indicates the rate of evidence accumulation over 7 different coherences. The most likely group means are plotted; error bars indicate the posterior standard deviation. With higher coherence, higher drift rates were observed for both OCD (red, n = 26) and control (blue, n = 44) subjects. However, at higher levels of coherence, OCD subjects exhibited significantly slower drift rates and reaction times than OCD subjects (q<0.05 for 30%, and 45% coherence; q<0.01 for 70% coherence). B) No significant differences in the posterior distribution of HDDM threshold were observed between OCD and control subjects.

Drift rate was calculated for each of seven ‘coherence’ levels (0.5%–70%) using HDDM in 26 OCD cases and 44 control participants who completed the RDMT in the no cost (first) condition. In a high uncertainty, low coherence (5% coherence) condition, no significant differences in modeled drift-rate was observed between OCD patients and controls. However, in the higher coherence (most certain) conditions, the OCD cases manifest significantly slower reaction times, and HDDM-modelled drift rates (Fig 3A). Both groups showed increasing drift rates as coherence increased, although this coherence-drift rate interaction was less dramatic for OCD cases. There was no significant difference between the two groups in the posterior distribution of the HDDM calculated thresholds (Fig 3B). These results replicate some of the previous findings distinguishing performance on the RDM task between OCD and non-OCD participants [9].

#### Comparison of high- and low-doubt participants

**Fig 4:**
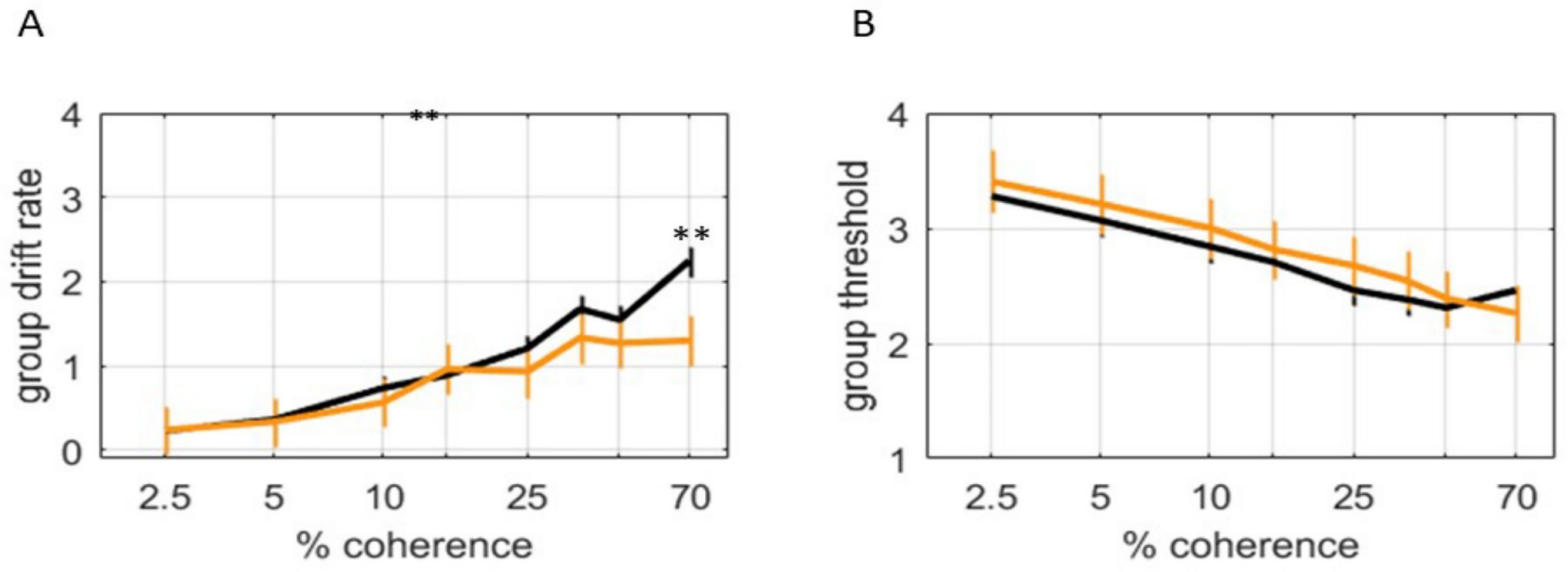
Behavioral task performance in participants with high (>60) and low (≤60) doubt scores. A) A posterior distribution of group mean drift rate was calculated using HDDM, and indicates the rate of evidence accumulation over 8 different coherences. The most likely group means are plotted; error bars indicate the posterior standard deviation. With higher coherence, higher drift rates were observed for both high doubt (orange color, N = 10) and low (black color, N = 36) subjects. However, at higher levels of coherence, high doubt subjects exhibited even faster drift rates and reaction times than low doubt subjects (q<0.01 for 70% coherence). B) No significant differences in the posterior distribution of HDDM threshold were observed between high and low doubt subjects.

We sought to investigate whether doubt might be a predictor of subjects’ RDM performance. We obtained doubt scores for a subset of the OCD and control participants included in the previous data. Individuals with high scores on the DQ (doubt score ≥60, N = 10) showed a significantly higher reaction time and lower modeled drift rate than participants with a low DQ (doubt score <60, N = 36) score, but only in the highest coherence (most certain) condition (Fig 4A). The difference in the number of participants in the low and high doubt groups may explain the lower statistical significance at other coherences, due to fewer number of subjects tested. The decision threshold, as in the OCD and control comparison, was not different between the groups (Fig 4B).

#### Relationship between doubt score and drift rate

**Fig 5:**
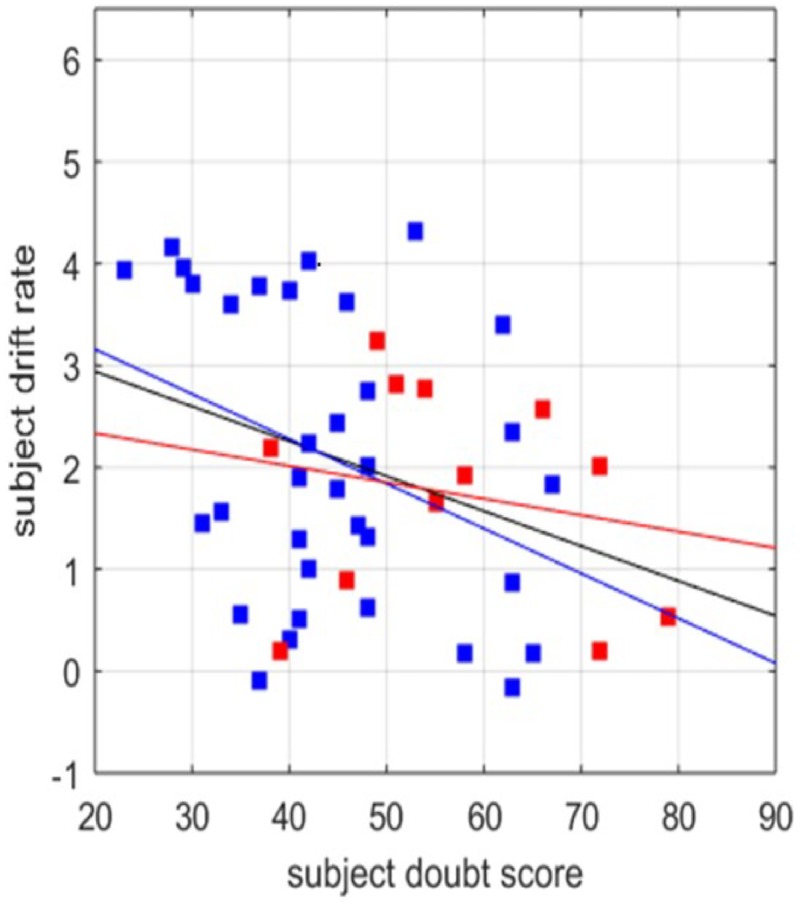
Relationship between doubt score and drift rate independent of OCD diagnosis. HDDM was used to calculate the most likely drift rate for each subject with both a doubt score and an OCD diagnosis. Comparisons of all subjects revealed a correlation between doubt score and drift rate at 70% coherence in the no cost condition (*v* = 3.63 − 0.034*DQ*; *R*^2^ = 0.11; *p* = 0.024. When subjects with OCD were excluded, control subjects (blue) also exhibited a correlation between doubt score and drift rate at 70% coherence in the no cost condition (*v* = 4.04 − 0.044*DQ*; *R*^2^ = 0.13; *p* = 0.038). In OCD subjects alone (red), there was not a significant correlation between doubt score and drift rate OCD subjects: *v* = 2.66 − 0.016*DQ*; *R*^2^ = 0.04; *p* = 0.53.

We modeled subjects’ individual thresholds and drift rates as a function of doubt score for all coherence levels, in participants who completed the DQ. There was a significant correlation between the doubt score and drift rate for all participants (r^2^ = 0.11; *p* <0.05) and for control participants (r^2^ = 0.13; *p* <0.05), exclusively at the 70% coherence level. We found no correlation between doubt score and threshold. The correlation between the drift rate and the doubt score was not significant for OCD cases alone, which may be attributable to the low number of OCD cases (N = 12) completing both the DQ and the RDMT, or heterogeneity among patients with OCD. However, the best-fit regression slopes between control and OCD subjects were not significantly different (*p* = 0.44) (Fig 5).

#### Relationship between doubt score and decision threshold in response to incentive

A posterior distribution of group mean decision thresholds was calculated, using HDDM, over five different coherence levels when subjects were incentivized to respond quickly (third condition). As reported above, under no cost conditions (first condition) there was no effect of coherence level on decision threshold; neither was there any effect of coherence on the threshold for OCD, control, or low doubt subjects. However, with higher coherence, high doubt subjects exhibited lower evidence thresholds than low doubt subjects (q<0.05 for 45% and 70% coherence levels).

#### Relationship between doubt score and self-reported certainty

In the second condition of the RDMT, participants reported the confidence with which they made their decisions with scores ranging from 1 (low confidence) to 7 (high confidence). The pooled distributions of confidence scores, based on correct trials, showed that high doubt subjects (n = 10) report less confidence than low doubt subjects (n = 36) when evidence accumulates more quickly, i.e. with the high coherence, less ambiguous tasks. We report the chi-squared statistic, which indicates that the higher coherence (25%, 45%, and 70%) distributions are significantly distinct between the two groups, <0.005 in each test. The 12 OCD subjects for whom we collected a doubt score exhibited less confidence than the 34 controls at all but the lowest coherence level, p<0.005.

## Discussion

Variability in the neurophysiological processes by which an individual accumulates information and directs his/her behavior may form the basis for individual differences in cognitive traits, and in some cases give rise to psychopathology. In this study, we focused on doubt, defined as a lack of subjective certainty or confidence in one’s perceptions, as a cognitive dimension linked to individual differences in computational parameters, the values of which define how external evidence for decision-making under uncertainty is accumulated.

To quantify doubt, we developed the DQ, and employed it in an internet sample and in the clinic. The DQ showed excellent psychometric properties, with doubt scores in the internet sample showing an approximately normal distribution, and factor analysis suggesting that a single factor underlies the doubt construct, with excellent inter-item reliability. External validation of the DQ was evidenced by a strong association of doubt scores with a self-completed assessment of the YBOCS doubt item (an item that does not presuppose the presence of obsessions). Moreover, doubt scores were significantly higher in OCD-affected than control participants, even after adjusting for age differences. Moreover, doubt was inversely associated with age in participants in both internet surveys, suggesting that the doubt score is not influenced by age-related memory decline.

We also found that doubt was associated with decision-making performance on the RDM task. Those with high doubt scores showed significantly slower reaction times and estimated drift rates than those with low doubt, under less uncertain conditions, although there was no effect on the computed decision threshold. Those with high doubt, moreover, reported less certainty in their responses. Together, these findings support both the validity of the DQ and a behavioral measure (evidence accumulation during perceptual decision-making) with which to assess individual variation in decision-making and doubt.

Self-report and behavioral approaches to measurement in behavioral research often lack agreement. This has been recognized, for example, in the measurement of other decision-making constructs and in ‘impulsivity’ [21, 22]. Therefore, the association between the self-report measure (DQ) and the behavior-based measure in this study is an important finding, providing confidence that they are assessing the same construct.

The pathological expression of doubt is considered a paramount feature of OCD. There is a long tradition of the clinical importance of doubt in OCD [23–27]. More recently, O’Connor’s research group has made important contributions to this body of work [28]. They postulate a cognitive process, termed inferential confusion, that is important in the origin of some obsessions, and they have developed a treatment modality (inference-based treatment (IBT) which aims to modify the reasoning style producing the obsessional doubt [29–31]. Our approach in the current paper differs, in that we propose an underlying trait, independent of the specific obsessional symptoms that involves decision-making in general, beyond the symptom itself.

The findings, comparing OCD participants and controls, are comparable to those comparing high and low doubt participants. OCD participants exhibit slower estimated drift rates than control participants, with no evidence of different decision thresholds. They also report lower certainty in their decisions than the controls. This provides further support for the presence of higher doubt measures in patients with OCD. A significant difference in drift rate between OCD subjects and controls in the highly coherent decision doubt and OCD are not equivalent, as the correlation between doubt scores and drift rate under low uncertainty conditions was observed even in those without OCD; moreover, the correlation between drift score and doubt score was greater for controls than for OCD participants. Participants with OCD exhibited a wide range of doubt scores, albeit typically in the higher range. Given the clinical heterogeneity of OCD, we suspect that doubt may contribute to the development of many but not all cases of OCD. Further prospective research is necessary for elucidating the contribution of doubt, and the interaction between doubt and other underlying traits, to the development of OCD.

Banca et al. [9] employed a similar behavioral paradigm comparing OCD cases and controls and found that individuals with OCD exhibited slower drift rates making decisions. Moreover, Banca et al. found that, in OCD cases, the decision threshold diminished under pressure of speed, a finding that was observed in high-doubt individuals in this study. This is consistent with the hypothesis that high doubt involves an inability to quickly update the decision threshold toward extremes. Under circumstances in which speed is rewarded, but the drift rate reaches a ceiling, lowered thresholds may be the individual’s only recourse to speed up reaction times, i.e. if the drift rate cannot be enhanced, to speed the decision process, the decision threshold would need to be lowered. In contrast to our finding regarding the decision threshold in OCD, Hauser et al. [11] found an increased threshold in high compulsive individuals. This difference may be explained by participant heterogeneity, in addition to different inclusion criteria. Nevertheless, further research is need to clarify this difference.

In this study, we found that the slower drift rate was most evident in the more coherent, less ambiguous choices, which is consistent with prior OCD studies [8]. We have extended the work of other investigators studying decision-making in OCD, by proposing that a dimensional trait, doubt, underlies some cases of OCD, but that it is neither a necessary nor sufficient factor in all cases of OCD.

The subjective reports of certainty reflect the presence of doubt in both the high doubt and OCD groups, particularly in the least ambiguous tests, and most strongly in the OCD comparisons. This was not observed in the findings reported by Banca et al. [9), but has been shown in other OCD studies [8, 32]. Interestingly, the report of reduced certainty in the decision-making may instead show that metacognitive deficits are more prominent in OCD, while the pattern of perceptual decision-making is well captured by doubt score alone. This is consistent with the findings of Hauser et al., which replicated the differences in perceptual decision-making between ‘compulsive” participants and controls, but also found additional differences in “metacognitive” uncertainty between them [10, 11].

What neurophysiological explanation could there be for limitations on the rates of evidence accumulation that we and others have observed? In the RDM task, the overall direction of the moving dots is uncertain. This assessment might be computationally conceptualized as an assignment of a probability (or belief) to each possible external state of the world. As new evidence occurs, these probabilities are updated in a Bayesian manner. When the probability profile, or belief state, reaches a relevant threshold, a behavior might be released or suppressed. In the RDMT, and in many choices in daily life, one’s belief state must be sufficiently near absolute certainty to release a behavior. Correspondingly, all other belief states must be sufficiently near 0. In more certain circumstances, evidence for one state accumulates quickly. In this situation, an efficient updating process would quickly suppress alternative beliefs to 0 while elevating the belief for the most-likely state towards 1. In a drift-diffusion model, the drift rate reflects this update rate. We find that high-doubt individuals exhibit slower drift rates at the highest levels of coherence in the RDM task, suggesting an inability to rapidly update belief towards more extreme values (0 or 1). When coherence is low, and uncertainty is high, evidence occurs slowly, and an update process with limited speed is not taxed. Reaction time also reflects the belief threshold required to release a behavior, which is reflected in the threshold measured by the drift diffusion model. An ideal threshold optimizes a speed-accuracy tradeoff.

The importance of the findings in this study is the correspondence between self-report questionnaire, behavioral measurement and a clinical syndrome OCD; each of these domains of assessment provide critical information relevant to understanding a condition. These different perspectives, together with several others, from the molecular to the neurophysiological, provide their own focus and contribution to the understanding of the condition.

Several potential limitations of the current study must be acknowledged. The internet sample of respondents may not be representative of the community, and the distribution of doubt scores in a more representative community sample must be examined. Moreover, the OCD case and control groups may not be representative of OCD cases and non-cases, respectively. Indeed, since the non-clinical comparison individuals were not evaluated for OCD, it is possible that some of them met criteria for a diagnosis of OCD, which would lead to under-estimation of the difference in doubt scores between OCD and non-OCD individuals. Although age and gender did not explain the higher doubt score in the OCD cases than control group, there may be other unmeasured demographic or clinical factors that might confound the relationship. It is important to acknowledge that HDDM is a hypothetical approach for understanding the process of decision-making, and alternative approaches may prove more appropriate. In future research, it would be useful to evaluate the encoding of sensory input, as distinct from the drift rate, and to determine whether doubt influences this process [33, 34].

## Conclusions

Future studies are needed to evaluate the DQ in a larger number of individuals, and in more representative, community and clinical samples, so that useful norms can be derived for clinical and research applications. Moreover, research is needed to determine how doubt scores are influenced by demographic characteristics, clinical features (e.g., Axis I comorbidity, personality traits), and life experiences. Of particular interest for OCD research is to determine if the severity of doubt is predictive of the development and severity of obsessions and compulsions, and to further explore whether therapeutic reduction of doubt can alter the course and severity of the disorder [28–31].

Further work also is needed to investigate the relationship of doubt to other psychiatric disorders, and the characteristics of apparently healthy persons who exhibit doubt. Determining the cognitive-behavioral and neurobiological underpinnings of the doubt dimension as an endophenotype for specific psychopathology should move the field further by taking advantage of the burgeoning understanding of perceptual decision-making and the neurocircuitry of decision-making, and may help to direct the development of more effective, rational treatments for OCD and other disorders.

## Supporting information

S1 Fig Scree plot results of factor analysis of Doubt Questionnaire, in phase one internet participants (N = 152). (PDF)

S2 Fig Classification of OCD status using Doubt Questionnaire doubt score, in OCD cases (N = 67) and non-OCD controls (N = 27). (PDF)

S1 Table Doubt Questionnaire. (PDF)
