# Supplementary material for: Validating a dimension of doubt in decision-making: A proposed endophenotype for obsessive-compulsive disorder
Source: PLoS One. 2019 Jun 13;14(6):e0218182. doi: 10.1371/journal.pone.0218182 (PMC6564001; doi:10.1371/journal.pone.0218182)

S1 Figure A. Scree plot results of factor analysis of Doubt Questionnaire, in phase one internet participants (N=152)

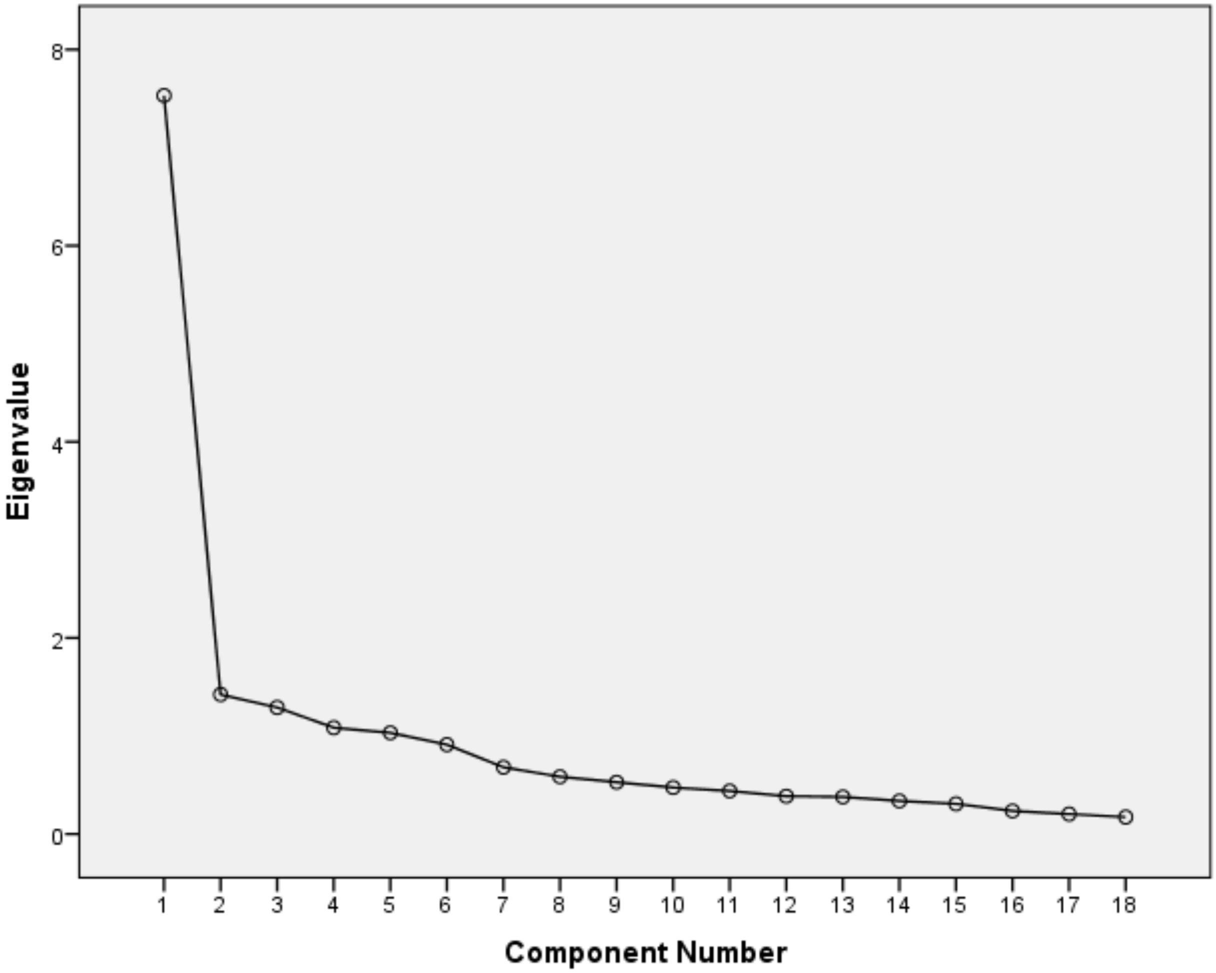

Supplement: S1 Fig — (PDF) [file pone.0218182.s001.pdf]
