# Supplementary material for: Validating a dimension of doubt in decision-making: A proposed endophenotype for obsessive-compulsive disorder
Source: PLoS One. 2019 Jun 13;14(6):e0218182. doi: 10.1371/journal.pone.0218182 (PMC6564001; doi:10.1371/journal.pone.0218182)
Supplement: S2 Fig — (PDF) [file pone.0218182.s002.pdf]

S2 Figure B. Classification of OCD status using Doubt Questionnaire doubt score, in OCD cases (N=67) and non-OCD controls (N=27)

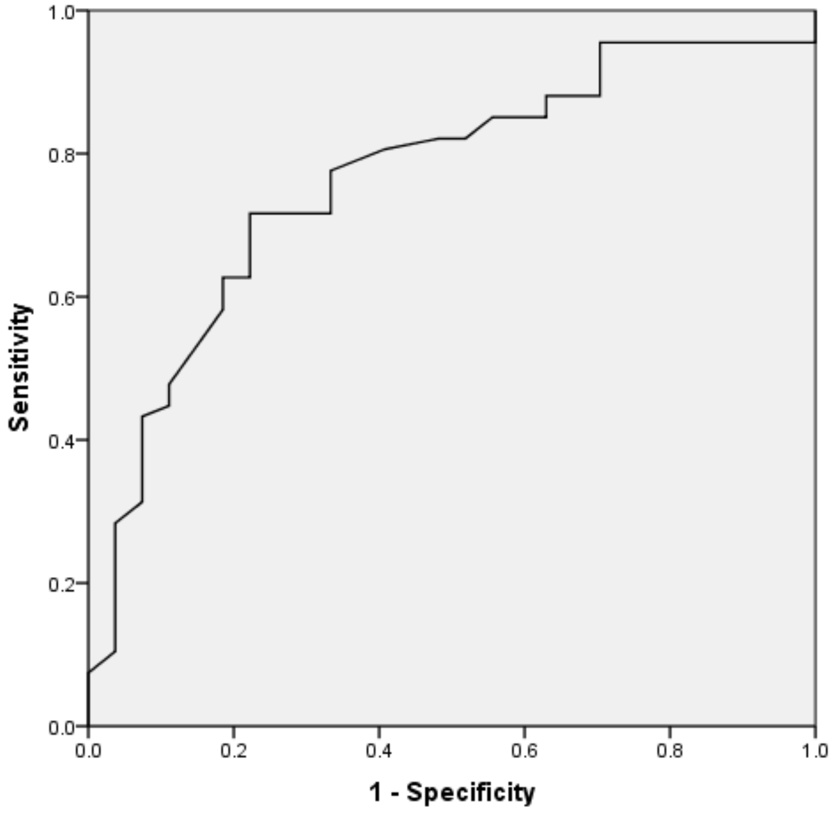

Area under the curve = 0.76 (95% CI=0.66-0.89), p<0.001.
