# Supplementary material for: Validating a dimension of doubt in decision-making: A proposed endophenotype for obsessive-compulsive disorder
Source: PLoS One. 2019 Jun 13;14(6):e0218182. doi: 10.1371/journal.pone.0218182 (PMC6564001; doi:10.1371/journal.pone.0218182)
Supplement: S1 Table — (PDF) [file pone.0218182.s003.pdf]

S1 Table A. Doubt Questionnaire

		Strongly disagree…………..…………..….Strongly agree				
1.	I need to reassure myself that something I did was actually completed.	1	2	3	4	5
2.	I am uncertain of my own recollections of what happened during an event.	1	2	3	4	5
3.	I trust my own intuition.	5	4	3	2	1
4.	When faced with a dilemma, it is difficult to know my own opinion.	1	2	3	4	5
5.	I question whether I'm sure of my facts.	1	2	3	4	5
6.	I feel that I might have missed something because I didn't look carefully enough.	1	2	3	4	5
7.	When I do mental arithmetic, I doubt my answer.	1	2	3	4	5
8.	When I count items in a list, I feel like I might have missed one.	1	2	3	4	5
9.	I don't trust my memory of simple, everyday things.	1	2	3	4	5
10.	Even when I'm pretty sure about a memory, I start to doubt whether it's really correct.	1	2	3	4	5
11.	I doubt my ability to remember accurately.	1	2	3	4	5
12.	I look at things a second time to be sure that I got an accurate look.	1	2	3	4	5
13.	It's easy for me to start doubting myself.	1	2	3	4	5
14.	It is difficult for me to skim through reading material because I doubt whether I really got the general idea.	1	2	3	4	5
15.	I have to read each individual word in a sentence.	1	2	3	4	5
16.	I second-guess my decisions.	1	2	3	4	5
17.	Even when I do something very carefully, I often feel that it is not quite done right.	1	2	3	4	5
18.	I usually have doubts about the simple everyday things I do.	1	2	3	4	5

Doubt score is the sum of the ratings for the 18 items. Note that item 3 is reverse coded.
